# Light-induced levitation of ultralight carbon aerogels via temperature control

**DOI:** 10.1038/s41598-021-91918-5

**Published:** 2021-06-14

**Authors:** Reo Yanagi, Ren Takemoto, Kenta Ono, Tomonaga Ueno

**Affiliations:** Department of Chemical Systems Engineering, Graduate School of Engineering, Nagoya University, Furo-cho, Chikusa-ku, Nagoya, 464-8603 Japan

**Keywords:** Carbon nanotubes and fullerenes, Porous materials

## Abstract

We demonstrate that ultralight carbon aerogels with skeletal densities lesser than the air density can levitate in air, based on Archimedes' principle, when heated with light. Porous materials, such as aerogels, facilitate the fabrication of materials with density less than that of air. However, their apparent density increases because of the air inside the materials, and therefore, they cannot levitate in air under normal conditions. Ultralight carbon aerogels, fabricated using carbon nanotubes, have excellent light absorption properties and can be quickly heated by a lamp owing to their small heat capacity. In this study, an ultralight carbon aerogel was heated with a halogen lamp and levitated in air by expanding the air inside as well as selectively reducing its density. We also show that the levitation of the ultralight carbon aerogel can be easily controlled by turning the lamp on and off. These findings are expected to be useful for various applications of aerogels, such as in communication and transportation through the sky.

## Introduction

The weight reduction of materials is crucial for building a resource- and energy-conscious society, realizing comfortable living spaces, and space exploration. Therefore, much research has been conducted on this topic. Aerogels^1^ and metallic microlattices^2,3^ are typical lightweight materials with large specific surface areas. As a result, they are attracting attention as structural materials as well as adsorbents^4,5^, energy storage materials^6^, catalyst support materials^7^, and thermal insulators^8^. To the best of our knowledge, the lightest material reported thus far is a ceramic aerogel with a density of 0.10 mg cm^−3^ ^9^. This is less than 1/10 of the density of air at room temperature (approximately 1.29 mg cm^−3^), and it is incredibly light. Owing to their high electrical, thermal, and mechanical properties, carbon aerogels have been studied for use in many applications^10–16^. Recently, research has also been conducted on the utilization of the light absorption and heating properties of such materials^17^.

The lightest carbon aerogel, discovered to date, has a density of 0.16 mg cm^−3^, as reported by Sun et al. in 2013^18^. The density of this aerogel is approximately 1/6 of the density of air at room temperature; however, it does not levitate in air. This is because there are many pores in the ultralight aerogel that are filled with air, and therefore the apparent density is the sum of those of the material and inner air, thereby making it heavier than air. Many studies have been conducted on ultra-lightweight materials^19–24^; however, the attempts to make them levitate in air have been very few and limited. If ultralight materials can levitate in air, they will behave as if they are defying gravity, providing new values to our lives. For example, Google's Loon project uses balloons that fly in the stratosphere to provide Internet access to remote areas where the Internet is not widely available. Materials that can spontaneously levitate in air can be applied to such technologies that involve transportation in air. Zhang et al. showed that graphene aerogel can be driven by electron emission when exposed to laser light in vacuum (6.7 × 10^–4^ to 9.1 × 10^–2^ Pa), and this mechanism was used to levitate graphene aerogel in a vacuum tube^25^. Azadi et al. reported that a centimeter-thick scale disk, made of 0.5 µm thick mylar film coated with carbon nanotubes (CNTs) on one side, levitates in a vacuum chamber (10–30 Pa) when irradiated by light. When the polymer disc is heated by light, the difference in the interactions of the upper and lower surfaces with the incident gas molecules acts as a driving force to levitate the disc^26^. Although these studies are interesting, the conditions for obtaining the driving force for levitation, such as a vacuum environment, are the limiting factors here. According to Archimedes' principle, if a material is lighter than air, then it should levitate because of the buoyancy of air, even when there is no other driving force.

The buoyant force (*F*) acting on an object in air is equal to the weight of air displaced by the object and can be expressed by the following equation:

1 $$F=\rho Vg,$$

where *ρ* is the density of air, *V* is the volume of the object, and *g* is the acceleration due to gravity. To determine whether an object levitates in air, we can compare this buoyant force with the weight of the object. Because the volume of air, displaced by the object, is equal to the volume of the object, we can simply compare their densities. If the density of the object is larger than that of air, then the object will sink in air, and if the density is smaller, then it will levitate in air. Furthermore, if the densities are equal, the object will be stationary in air.

**Figure 1:**
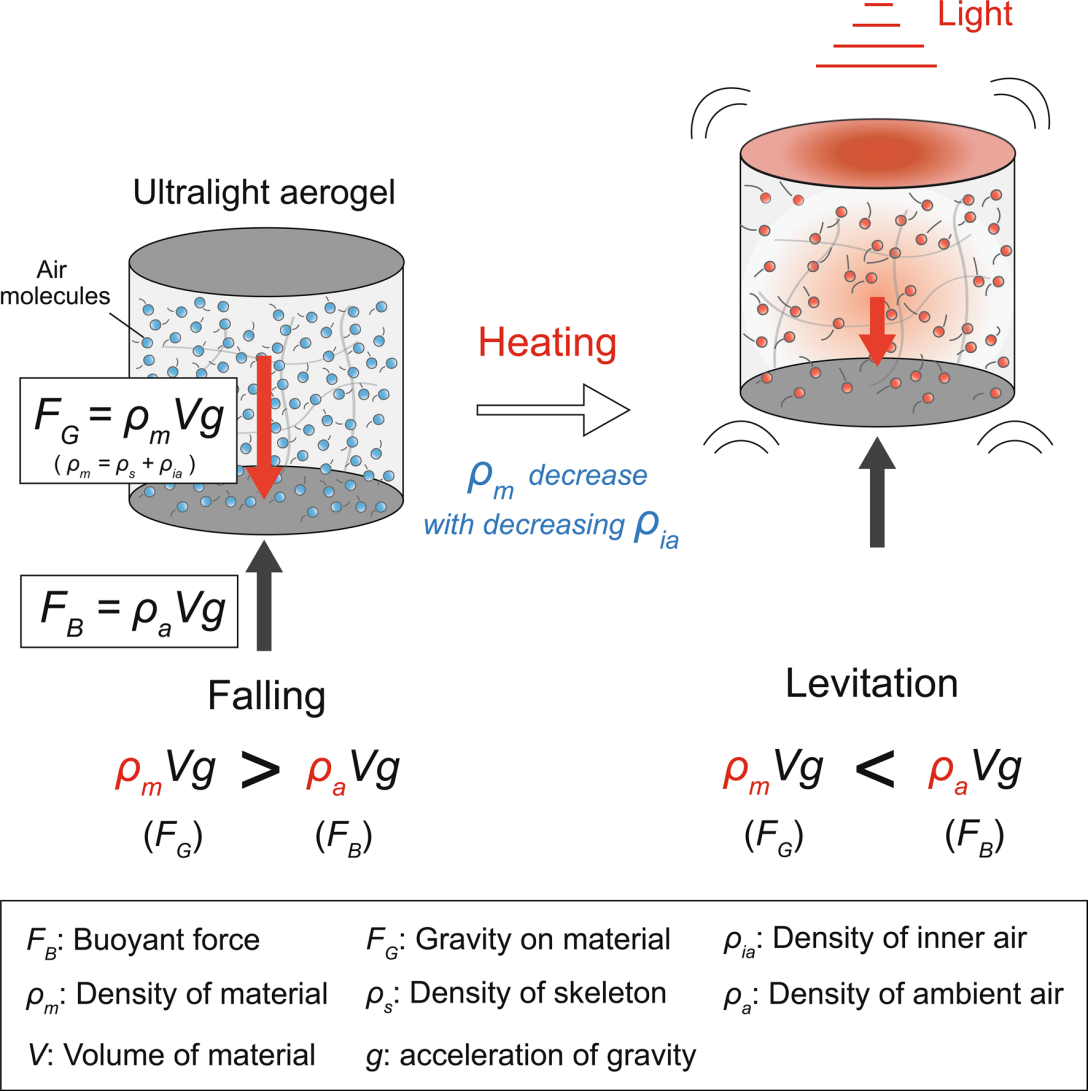
Levitation of ultralight aerogel.

In this paper, we report ultralight carbon aerogels that can intermittently levitate in air for a long time according to Archimedes' principle. Ultralight carbon aerogel fabricated with CNTs can be easily heated by light, and the temperature can be controlled. A carbon aerogel, with density that is less than that of air, is heated using a halogen lamp. As a result, the air inside expands to reduce the air density inside the aerogel, thereby creating a state in which the sum of the densities of the aerogel and inner air is less than that of the surrounding air. This allows the material to levitate due to buoyancy (Fig. 1). In addition, owing to its small heat capacity, the temperature inside the aerogel can be changed instantaneously by turning the lamp on and off, indicating that the levitation of the aerogel can be controlled using this phenomenon.

## Results

### Structural analysis of materials and various properties

Ultralight aerogels, composed of single-walled carbon nanotubes (CNTs) and cellulose nanofibers (CNFs), were produced by a freeze-drying process. We prepared ultralight aerogels with a prepared density of 0.25, 0.50, 0.75, and 1.0 mg cm^−3^ and named the samples as ULM 0.25, ULM 0.50, ULM 0.75, and ULM 1.0, respectively. CNFs were used to disperse CNTs in water. Because CNTs and CNFs are fibers with high aspect ratios, they bundle and intertwine well to form a lightweight framework, thereby enabling the fabrication of aerogels that retain specific mechanical properties in the ultralight density range below that of air.

**Figure 2:**
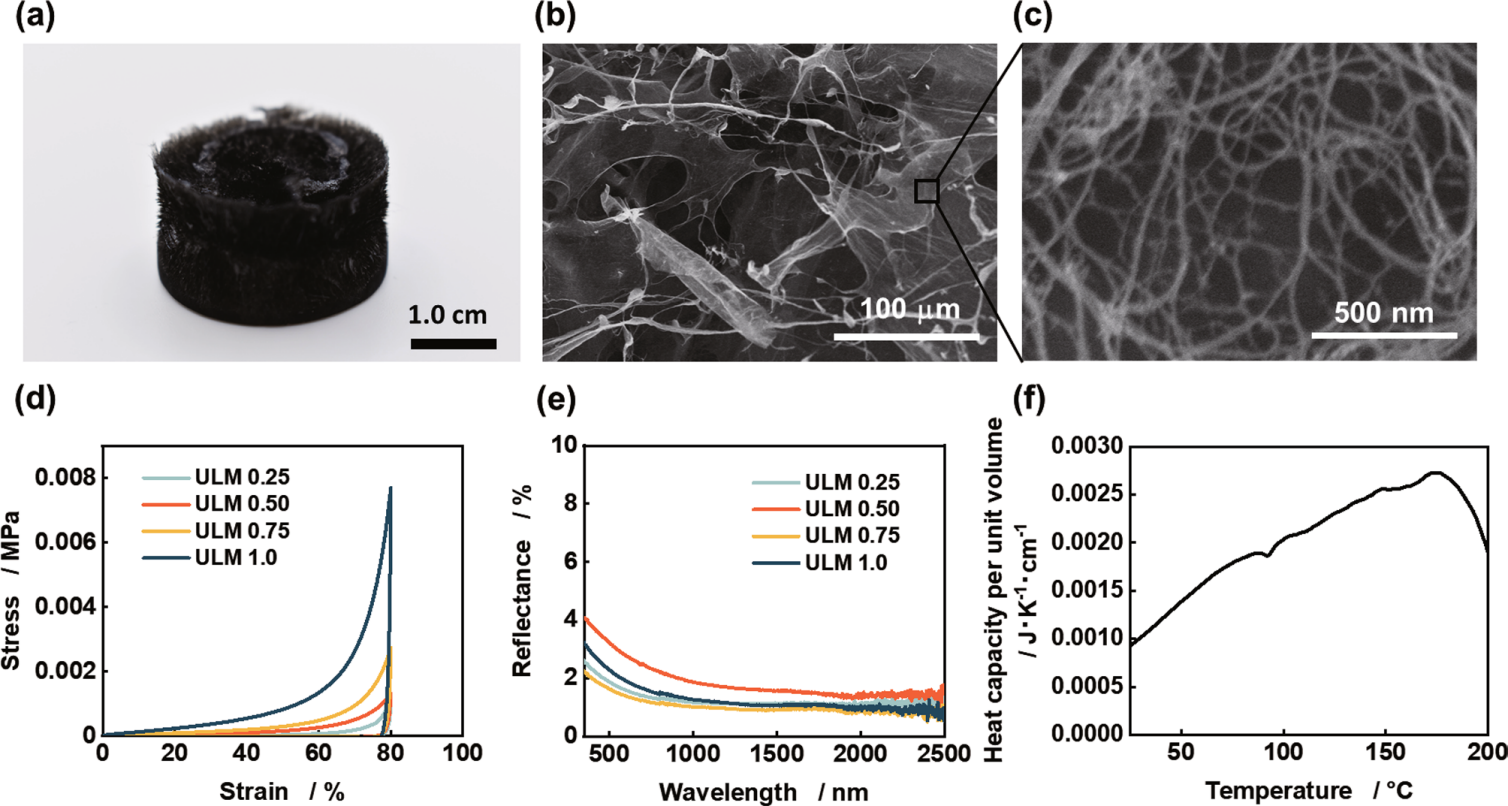
Material characterization of the ultralight aerogels. (**a**) Optical image of the aerogel. (**b**) SEM image at low magnification (×400). (**c**) SEM image at high magnification (×80,000). The optical and SEM images are of ULM 0.75. (**d**) Stress–strain curves of the ultralight aerogels fabricated by varying the prepared density. (**e**) Diffusive reflectance spectra of the ultralight aerogels fabricated by varying the prepared density. (**f**) Heat capacity per unit volume of ULM 1.0.

The microstructure, mechanical properties, light absorption properties (obtained by diffused reflectance measurements), heat capacity per unit volume, and an optical image of the fabricated aerogel are shown in Fig. 2. The fabricated aerogel was black and appeared as a cylindrical sponge that did not transmit visible light (Fig. 2a). The scanning electron microscopy (SEM) images show that the fabricated aerogel had a pore structure consisting of thin walls of intertwined fibers with a diameter of 26 ± 0.84 nm (Fig. 2b,c). Because the diameter of the CNTs was approximately 1–3 nm and that of the CNFs was approximately 3 nm, the fibers that composed the wall were a mixture of intertwined CNTs and CNFs. The prepared aerogels contained mesopores and macropores with diameters ranging from 2 to 250 nm and micropores with diameters ranging from 1.8 to 2.0 nm. The specific surface areas of all the samples ranged from 164 to 186 m^2^ g^−1^ and have similar microstructures regardless of the density (see Supplementary Fig. S5 online). The stress–strain curves obtained from the compression tests showed that the elastic moduli at 20% strain were 0.020, 0.20, 0.50, and 1.2 kPa for the samples ULM 0.25, ULM 0.50, ULM 0.75, and ULM 1.0, respectively, and this stress increased linearly up to a strain of approximately 60% (Fig. 2d). The stress increased rapidly with further strain, and the ULM 1.0 sample showed a pressure of 7.7 kPa at 80% strain. The gradual increase in the stress, observed for approximately 60% strain, is attributed to the collapse of voids in the aerogel. The subsequent rapid increase in stress is because of the compression of the framework forming the aerogel. Even in a very low-density aerogel, CNTs and CNFs, which have excellent mechanical properties, are intertwined and well dispersed within the aerogel; consequently, the material shows a mechanical strength enough to maintain structural integrity of the material.

UV–visible/near-infrared spectroscopy (UV–vis/NIR) measurements using an integration sphere^27^ show that the diffuse reflectance of all samples is between 0 and 4% in the wavelength range of 350–2500 nm (Fig. 2e). These measurements were performed on samples of 5 mm thickness. The transmittance in the same measurements is less than 5% for ULM 0.25 (see Supplementary Fig. S7 online), and for the other samples the transmittance is smaller due to the larger density. Thus, the fabricated ultralight aerogel absorbs more than 90% of light in the wavelength range of 350–2500 nm and has high light absorption properties.

Figure 2f shows the heat capacity per unit volume of ULM 1.0. This was determined by multiplying the specific heat (ranging from 2.7 to 0.23 J g^−1^ K^−1^) that was obtained by differential scanning calorimetry (DSC) by the prepared density of the aerogel (g cm^−3^). The ultralight aerogel fabricated in the temperature range of 20–200 °C showed a heat capacity of approximately 9.3 × 10^–4^ to 2.7 × 10^–3^ J K^−1^ cm^−3^ per unit volume, which tended to increase with temperature. The small heat capacity across the entire temperature range suggests that the aerogel could be heated instantaneously using a small amount of energy. These small heat capacities can be attributed to the low density of the ultralight aerogel's skeleton. The other samples (ULM 0.25, ULM 0.50, and ULM 0.75) had smaller densities, and accordingly, their heat capacity per unit volume was also smaller (see Supplementary Fig. S8 online).

### Experimental results of ultralight aerogel levitation

**Figure 3:**
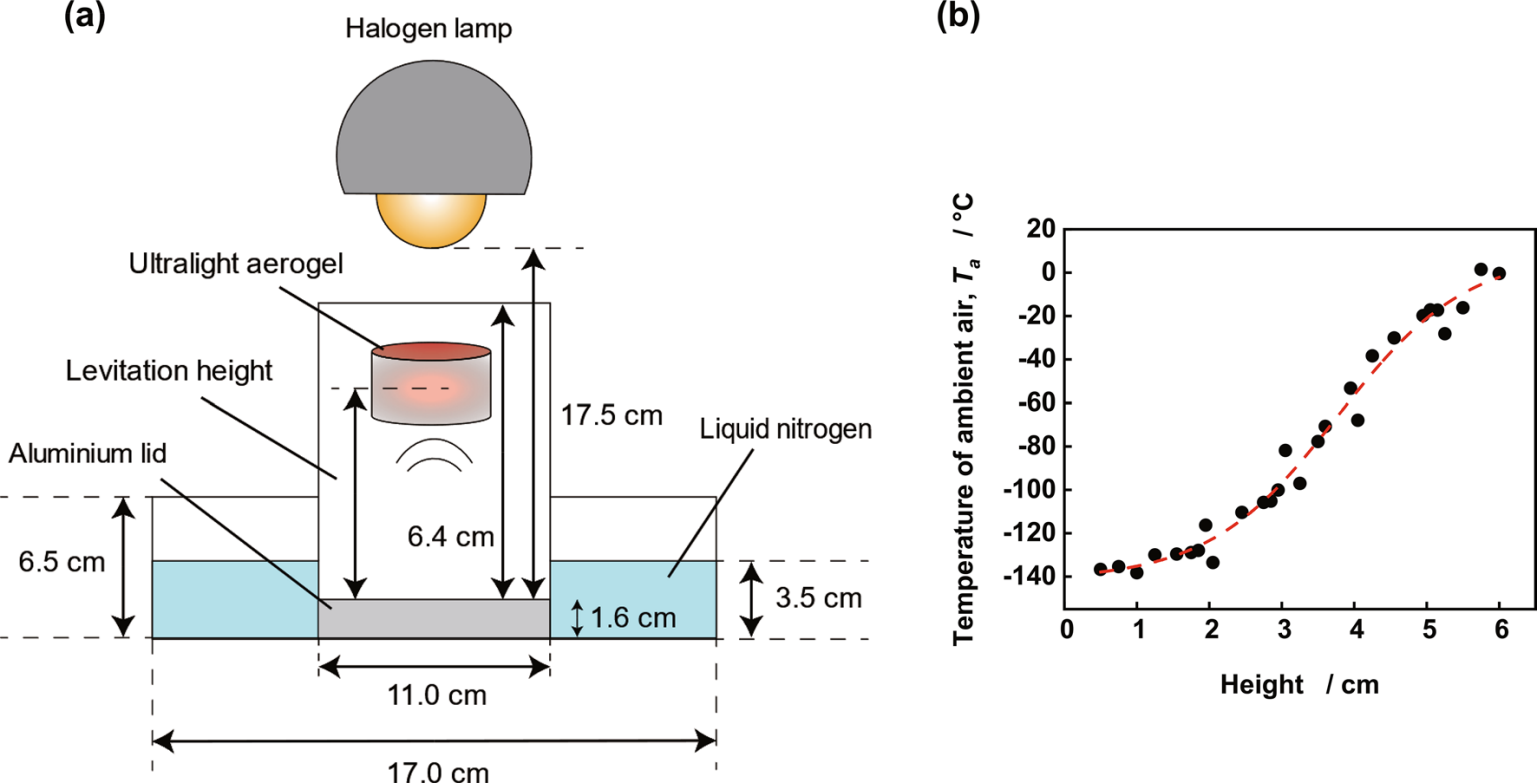
Setup of the levitation experiment. (**a**) Schematic diagram of the levitation experiment. (**b**) Temperature distribution in the cylinder during the levitation experiment.

**Figure 4:**
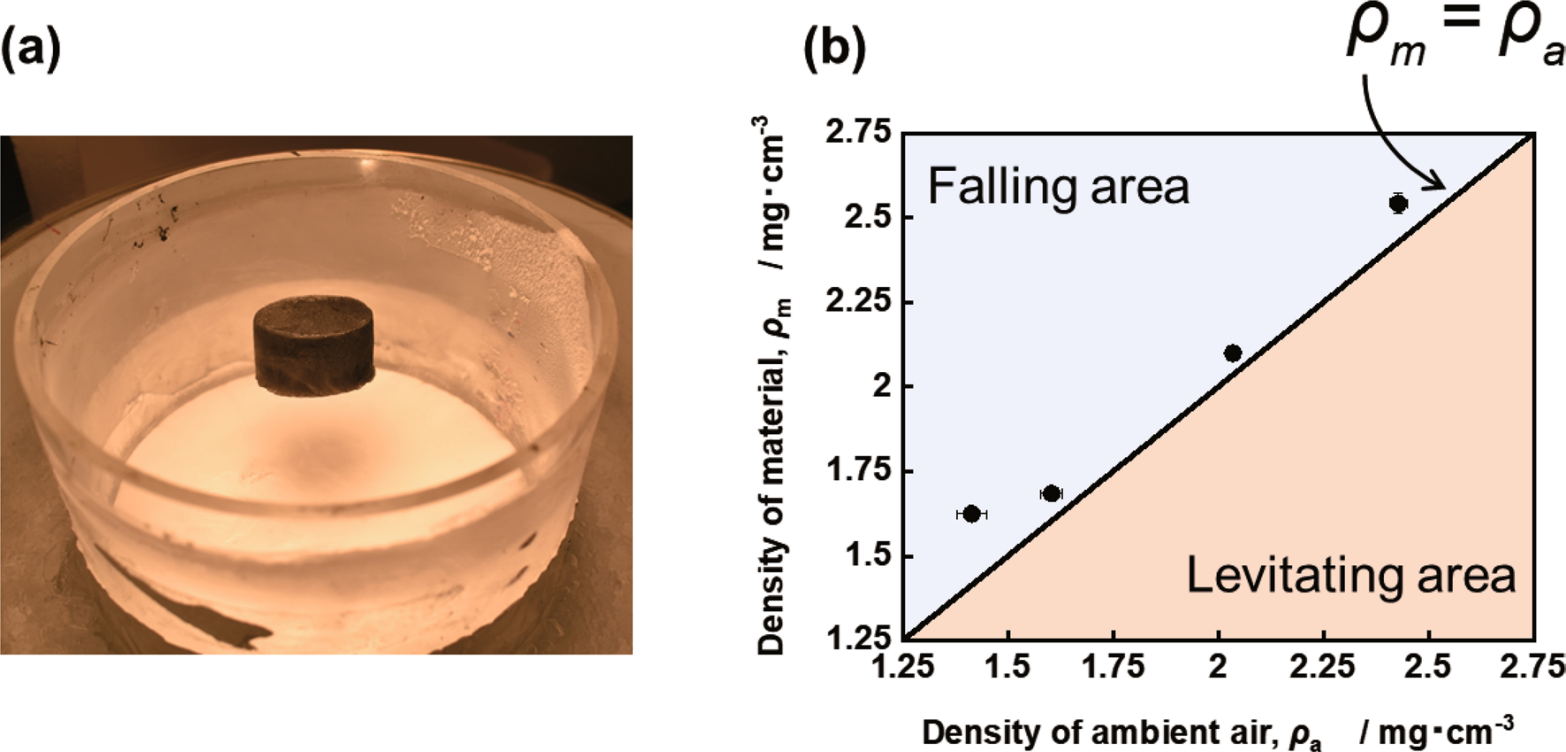
Results of the levitation experiments with ultralight aerogels of various densities. (**a**) Optical image of an aerogel during levitation. (**b**) Relationship between the density of the sample during levitation and that of ambient air. The ultralight material levitates in the range where *ρ*_*a*_ > *ρ*_*m*_. (The plots are the average of three measurements, and the error bars represent standard error).

The experimental set-up for levitation of ultralight aerogel is shown in Fig. 3a. The levitation experiment was conducted by heating the ultralight aerogel with a 90 W halogen lamp in an acrylic cylinder whose bottom was covered with an aluminum lid placed in a container filled with liquid nitrogen. As the bottom of the acrylic cylinder was covered with an aluminum lid, the liquid nitrogen did not enter the cylinder, and the thermal conductivity of the bottom was good. Figure 3b shows the temperature distribution of the ambient air temperature in the acrylic cylinder during the levitation experiment, as measured by thermocouples. The temperature distribution in the cylinder was measured after liquid nitrogen was poured into the cylinder and maintained for 5 min with the lamp on. The red dashed line in Fig. 3b is an approximate curve obtained by fitting the obtained temperature distribution with the Boltzmann function. Figure 4 and Table 1 summarize the results of the aerogel levitation experiments. When the lamp was turned on, the aerogel was heated and began to levitate (Fig. 4a). A cylinder surrounded the aerogel, and the bottom was covered with an aluminum lid such that it was not affected by the updraft caused by the liquid nitrogen. The aerogel levitated as long as it remained exposed to light from the lamp (see Supplementary Video S1 online). The levitating aerogel behaved as if it was levitating on top of the water, and when touched, the resistance due to buoyancy was perceived (see Supplementary Video S2 online). Table 1 shows the levitation height and the aerogel temperature (*T*_*m*_) for each sample during levitation as measured by a thermograph. When the sample was changed to ULM 0.25, ULM 0.50, ULM 0.75, and ULM 1.0, the levitating height also changed to 5.0, 4.1, 2.9, and 1.7 cm, respectively. The temperature of the aerogel during the levitation also changed to 151, 127, 80, and 45 °C, respectively. Since the levitation height of the aerogel is affected by the slight airflow present in the cylinder, and *T*_*a*_ is affected by the positional relationship with the lamp, the levitation height and *T*_*a*_ were measured three times for each sample, and the average values are shown in Table 1. Because the heat capacity of air is small, the air temperature inside the aerogel should be equal to these temperatures. The temperature of the aerogel increased as the aerogel density decreased because the lower the density of the material, the higher the balancing position and the closer it is to the lamp.

**Table 1:** Levitation height of each sample, temperature during levitation, temperature of ambient air, density of the skeleton of the samples, density of the air inside the samples, density of the samples, and density of ambient air (The values are the average of three measurements).

Sample	Height	*T*_*m*_	*T*_*a*_	*ρ*_*s*_	*ρ*_*ia*_	*ρ*_*m*_ (= *ρ*_*s*_ + *ρ*_*ia*_)	*ρ*_*a*_
	[cm]	[°C]	[°C]	[mg cm^−3^]	[mg cm^−3^]	[mg cm^−3^]	[mg cm^−3^]
ULM 0.25	5.0	151	− 23.2	0.79	0.83	1.62	1.41
ULM 0.5	4.1	127	− 53.0	0.80	0.88	1.68	1.60
ULM 0.75	2.9	80	− 99.7	1.10	1.00	2.10	2.04
ULM 1.0	1.7	45	− 127.8	1.44	1.11	2.54	2.43

*T*_*m*_: Temperature of materials.

*T*_*a*_: Temperature of ambient air.

*ρ*_*s*_: Density of skeleton with moisture.

*ρ*_*a*_: Density of ambient air.

*ρ*_*ia*:_ Density of inner air.

*ρ*_*m*:_ Density of material.

To confirm that the ultralight aerogel was heated by the halogen lamp and levitated via buoyancy according to Archimedes' principle (as shown schematically in Fig. 1), the densities of the aerogel and ambient air during levitation were calculated and compared. Table 1 shows the ambient air temperature (*T*_*a*_) in the cylinder during the levitation experiment. These values were obtained from the height at which each material levitated using the approximate curve of the temperature distribution in the cylinder in Fig. 3b. Table 1 also summarizes the density of the ambient air (*ρ*_*a*_) at each height, calculated from *T*_*a*_ using the ideal gas equation of state and the aerogel density during the levitation (*ρ*_*m*_). *ρ*_*m*_ is calculated as the sum of the skeleton density of the material (*ρ*_*s*_) and the density of air in the aerogel (*ρ*_*ia*_). The aerogel absorbs ambient moisture during the levitation experiment, which slightly increases the density of the skeleton. Therefore, *ρ*_*s*_ was measured after the levitation experiment. The error of *ρ*_*s*_ is less than ± 0.05 mg cm^−3^ for three measurement. *ρ*_*ia*_ is calculated from the temperature of the material during levitation using the ideal gas equation of state. The balance between the buoyant force of the ambient air and the gravity on the ultralight aerogel can be expressed by Archimedes' principle as follows:

2 $${\rho }_{a}Vg={\rho }_{m}Vg,$$

where *V* is the volume of the ultralight aerogel. After removing *V* and *g* from Eq. (2), the balance of forces is given by the following equation:

3 $${\rho }_{a}={\rho }_{m}$$

When *ρ*_*a*_ > *ρ*_*m*_, the ultralight aerogel can levitate via buoyancy (Fig. 1); the aerogel levitates in air at the position where *ρ*_*a*_ = *ρ*_*m*_. Figure 4b shows the relationship between *ρ*_*a*_ and *ρ*_*m*_. The plots are the average of the values calculated from the three measurements, and the error bars represent standard error of *ρ*_*a*_ and *ρ*_*m*_ respectively. All the plots obtained from the calculation results are near the line where *ρ*_*a*_ = *ρ*_*m*_, indicating that air buoyancy levitates the ultralight aerogel, according to Archimedes' principle, when heated by the halogen lamp. As mentioned before, the bottom of the acrylic cylinder was covered with an aluminum lid so that the updraft of liquid nitrogen does not affect the measurements. The value for ULM 0.25 is farther from the balanced position than that for the other samples because the density of ULM 0.25 is much less than that of the other samples, and thus it is more susceptible to the slight airflow present in the cylinder.

### Levitation of ultralight aerogels by turning a lamp on and off

**Figure 5:**
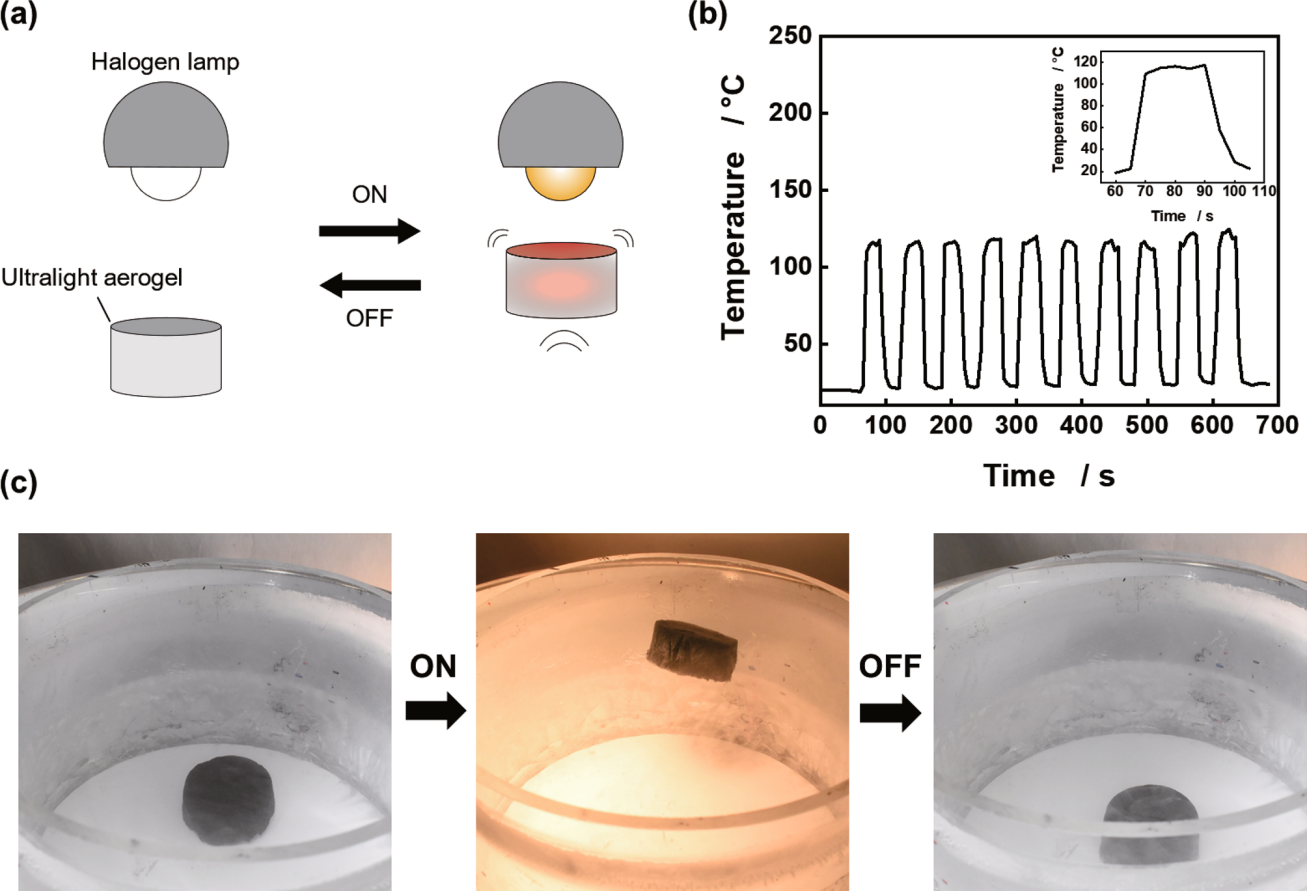
Results of the levitation experiments by turning a lamp on and off. (**a**) Schematic diagram of the levitation experiment with the ON–OFF cycling of the lamp. (**b**) Temperature response of the ultralight aerogel to the ON–OFF cycling of the lamp; inset: zoomed-in view of temperature variation in the range of 60–105 s. The data were obtained from ULM 0.75. (**c**) Optical image of the response of the ultralight aerogel to the ON–OFF cycling of the lamp.

Because of the light absorption property and small heat capacity of the ultralight aerogels shown in Fig. 2, the ultralight aerogels are instantly heated and levitate when the halogen lamp is turned on, and they instantly return to room temperature and fall to the ground when the lamp is turned off (see Supplementary Video S3 online). Considering this phenomenon, we conducted cyclic tests in which the halogen lamp was repeatedly turned on and off (Fig. 5a). These experiments were conducted using ULM 0.75. Figure 5b shows the temperature of the ultralight aerogel in response to the ON–OFF cycling of the halogen lamp. In this cycling test, the ultralight aerogel was placed on an aluminum foil and the temperature change was measured using a thermocouple attached to the top of the aerogel. The lamp was placed at a height of 17.5 cm from the bottom, same as that in the levitation experiment, and the ON (30 s)-OFF (30 s) cycle was repeated ten times. When the lamp was turned on, the ultralight aerogel was instantly heated to approximately 115 °C (Heating rate: approximately 17 °C s^−1^), and when the lamp was turned off, it instantly returned to room temperature (Cooling rate: approximately −12 °C s^−1^). Figure 5c shows the levitation behavior of the aerogel realized via the ON (10 s)-OFF (5 s) cycling test. Over repeated cycles, the ultralight aerogel responded agilely to the lamp ON and instantaneously rose to similar heights (see Supplementary Fig. S9 online). The ultralight aerogel began to fall when the lamp was turned off. This result indicates that the ultralight aerogel levitates owing to its light absorption property and small heat capacity. The light absorption performance of the ultralight aerogel is attributed to the excellent light absorption properties of CNTs owing to the optical transitions of π-electrons^28^. In addition, previous research has shown that the micropore (with a size smaller than 2 nm) may reduce the mean free path of electrons in the conductor^27^ and promote light absorption and increase the thermal equilibrium temperature of the aerogel (hot electron effect)^29^. Since the aerogels in this study also contain micropores with diameters ranging from 1.8 to 2.0 nm (see Supplementary Fig. S5 online), this hot electron effect may promote the high light absorption and photothermal conversion properties of the aerogels.

CNTs also absorb a range of wavelengths of sunlight, and research on the use of sunlight on CNT aerogels is currently underway^17^. The ultralight aerogel fabricated in this study can be levitated using actual sunlight (see Supplementary Video S4 online).

## Conclusion

In summary, we fabricated an ultralight aerogel with density less than that of air by compositing CNTs and CNFs and demonstrated that it can be levitated in air by controlling the temperature (which reduces the air density inside the aerogel). The fabricated ultralight aerogel could be instantly heated using a halogen lamp owing to its high light absorption property and small heat capacity. The ultralight aerogel heated by the lamp was levitated by the buoyancy of the surrounding air, and the levitation behavior could be controlled by the on/off cycling of the light source. Research on the levitation of CNT aerogels using sunlight is ongoing. The ultralight aerogel, which can be easily levitated by light, is expected to have a wide range of applications. If we can fabricate an aerogel of extremely large size, it can be theoretically used to transport people and objects via levitation. Furthermore, if such an aerogel can absorb sunlight efficiently, then it will significantly expand the possibilities of humanity's use of air as a resource, such as installing renewable energy power plants and radiowave sources floating in the air, and the development of air-based means of transportation, such as flying carpets.

## Methods

### Material

CNTs (eDIPS EC 2.0, Meijo Nano Carbon Co., Ltd.) with diameters of 1–3 nm and lengths of 1 µm or more were used to fabricate the composite aerogels. Cellulose nanofibers (RHEOCRYSTA I-2SX, DKS Co., Ltd.) with a diameter of 3 nm were used as the CNFs.

### Fabrication of ultralight aerogels

The CNT and CNF components were added to distilled water containing 1% ethanol. The sample compositions were adjusted to 0.25, 0.5, 0.75, and 1.0 g L^−1^ to fabricate composites with prepared densities of 0.25, 0.50, 0.75, and 1.0 mg cm^−3^, respectively. The CNT mass ratio was 70%. The dispersions were ultrasonically agitated for 12 min using an ultrasonic homogenizer (Sonifier 450, Emerson Japan Ltd.). These dispersions were cooled at 5 °C for 30 min in a low-temperature water bath and were subsequently frozen at -80 °C for 2 h in a small ultralow temperature bath (VT-78, Nippon Freezer Co., Ltd.). Finally, the dispersions were lyophilized (FDU-12AS freeze dryer, As One Corporation). Low-density CNT/CNF aerogels were fabricated using the above-mentioned process. According to the density of each sample, they were labeled as ULM X, where X = 0.25, 0.50, 0.75, or 1.0 according to the prepared densities of the aerogels. All samples were prepared with a diameter of 2.5 cm and a height of 1.5 cm. The actual densities of ULM 0.25, ULM 0.50, ULM 0.75, and ULM 1.0 were 0.25 ± 0.04, 0.52 ± 0.01, 0.84 ± 0.02, and 1.24 ± 0.04 mg cm^−3^, respectively, as determined by weight measurements after sample preparation. (These values are the average of three measurements and standard error).

### Levitation experiment

An acrylic cylinder (diameter: 11.0 cm), covered at the bottom with an aluminum lid, was placed in a cylinder (diameter: 17.0 cm). Liquid nitrogen was poured into the cylinder, with a diameter of 17.0 cm, to a height of 3.5 cm and maintained for 5 min. The ultralight aerogel was then placed inside the acrylic cylinder and heated with a 90 W halogen lamp (central luminous intensity: 2400 cd, beam angle: 30°, beam luminous flux: 580 lm) (ELPA, EBRF110V90W). During the experiment, the height of the halogen lamp was fixed at 17.5 cm from the bottom of the cylinder. The levitation height was measured as the distance from the bottom of the cylinder to the center of the levitating aerogel. The temperature of the ultralight aerogel during the levitation was measured by thermography. The experiment was performed three times for each sample, and the average value was calculated.

## Supplementary Information

Supplementary Information 1.

Supplementary Video 1.

Supplementary Video 2.

Supplementary Video 3.

Supplementary Video 4.

## Data Availability

The data that support the findings of this study are available from the corresponding author upon reasonable request.
