# Supplementary material for: Light-induced levitation of ultralight carbon aerogels via temperature control
Source: Sci Rep. 2021 Jun 14;11:12413. doi: 10.1038/s41598-021-91918-5 (PMC8203743; doi:10.1038/s41598-021-91918-5)
Supplement: Supplementary file 1 — Supplementary Information 1. [file 41598_2021_91918_MOESM1_ESM.pdf]

Supplementary Information

Light-induced levitation of ultralight carbon aerogels via temperature control

Reo Yanagi, Ren Takemoto, Kenta Ono, Tomonaga Ueno*

Department of Chemical Systems Engineering, Graduate School of Engineering,
Nagoya University
Furo-cho, Chikusa-ku, Nagoya 464-8603, Japan

*E-mail: ueno.tomonaga@material.nagoya-u.ac.jp

Material characterization and instruments

Surface structure

The surfaces of the samples were examined using field-emission scanning electron microscopy (FESEM; S4800, Hitachi High-Technologies Corporation).

Mechanical properties

The mechanical properties were measured via compression tests using a universal testing instrument (AGS-5kNX, Shimadzu). The elastic modulus at 20 % strain was measured from the obtained stress–strain curves. The crosshead speed during the test was 1 mm/min. The test samples were prepared each with a diameter of 2.5 cm and height of 1.5 cm.

Specific heat

The specific heat of the materials was measured using differential scanning calorimetry (DSC; DSC-60A, Shimadzu). The DSC measurements were performed at a heating rate of 99 °C min^{-1} over a temperature range of 20–200 °C.

Pore size distribution and specific surface area

The pore size distribution and specific surface area were measured by using an N_2 adsorption analyzer (TriStar II, micrometrics®) using the Brunauer–Emmett–Teller nitrogen adsorption/desorption technique.

Reflectance and transmittance[1,2]

The reflectance spectra of the samples were measured by a UV–Visible/NIR spectrometer (V-570DS, JASCO Corporation; here, UV is ultraviolet, NIR is near infrared), equipped with an integration sphere, in the wavelength range of 350–2500 nm. Spot size of the incident beam was approximately 8 × 9 mm for reflectance and 7 × 4 mm for transmittance. The thickness of the test sample was approximately 5 mm.

X-ray diffraction (XRD) Pattern

XRD measurement of the samples were carried out on a Rigaku SmartLab system using Cu Kα radiation.

Density calculation of air

The state equation for determining the air density (ρ) of an ideal gas is as follows:

$$\rho = \frac{PM}{RT} \tag{1}$$

Here, $P = 101325\ [Pa]$, $M = 28.966\ [g\ mol^{-1}]$, and $R = 8.314\ [Pa\ m^3\ K^{-1}\ mol^{-1}]$.

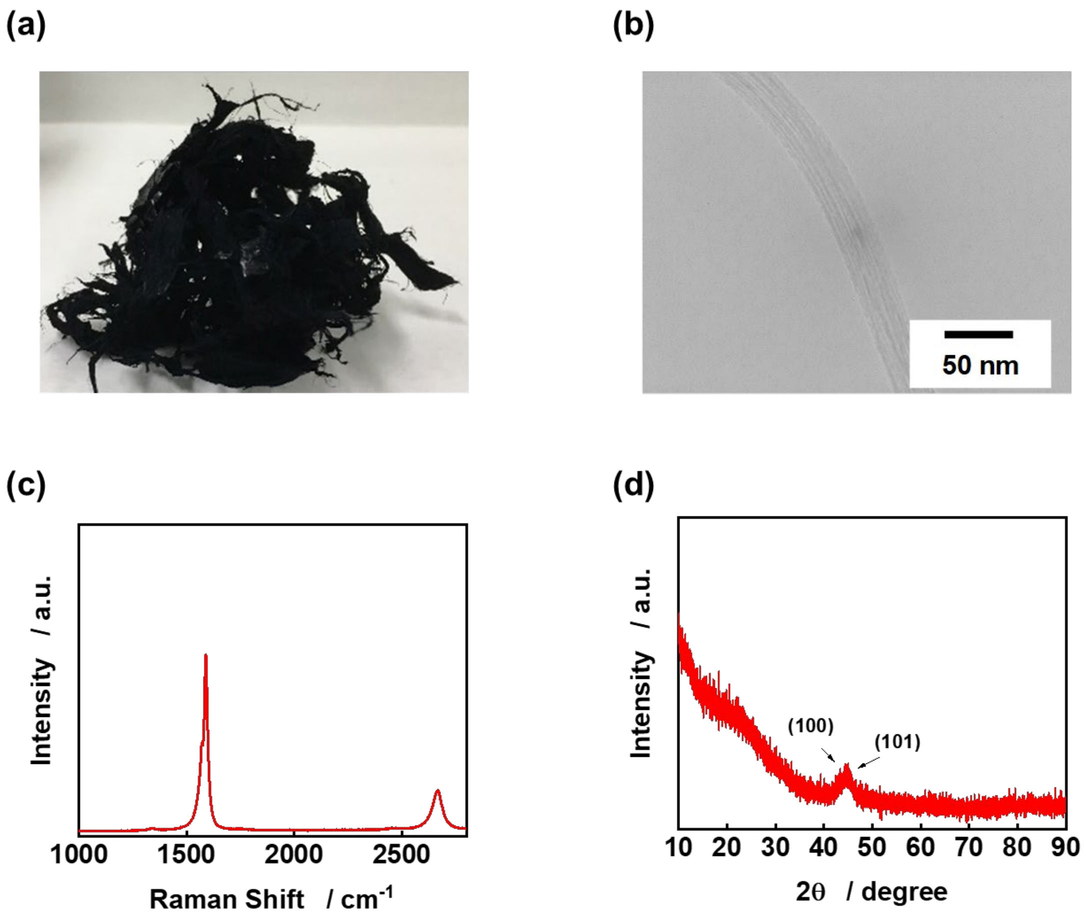

Figure S1. Characterization data of CNT used in this study. (a) Optical image. (b) Transmission electron microscope (TEM) image of bundled CNT. (c) Raman spectrum. (d) XRD pattern.

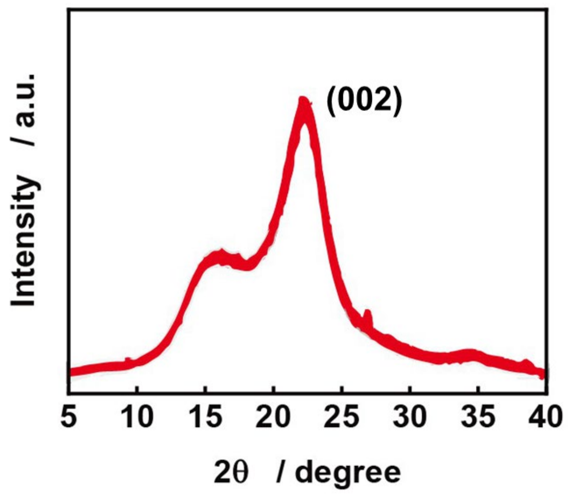

Figure S2. XRD pattern of CNF used in this study (The data is adopted from the reference[3]).

The TEM image of the CNF used in this study can be found on the referenced website[4].

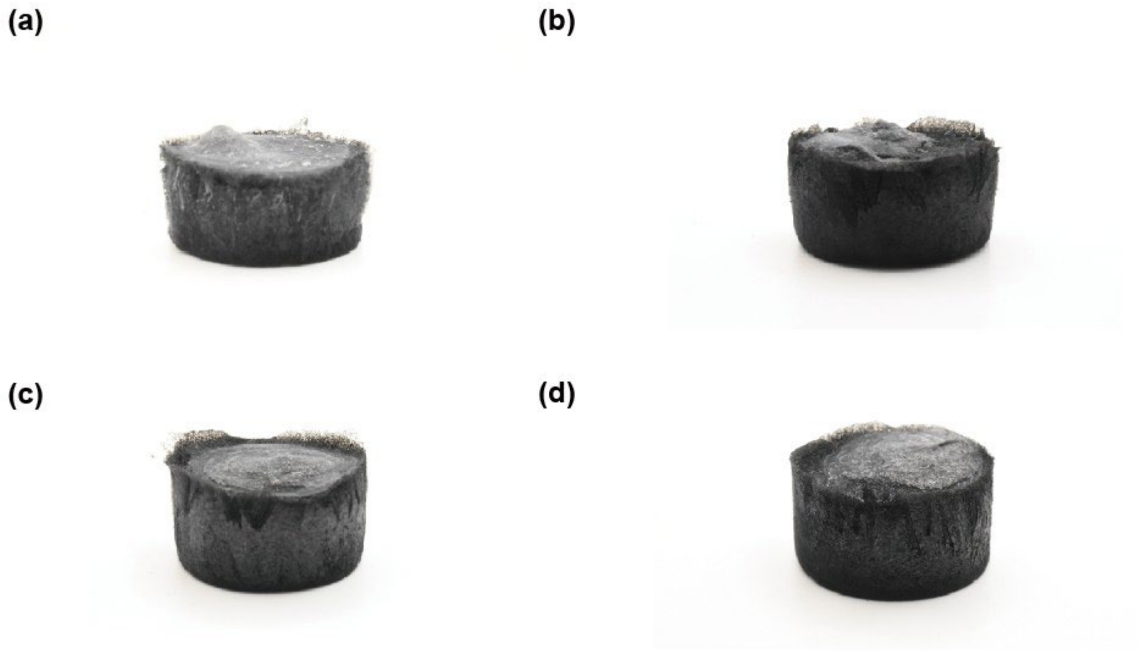

Figure S3. Optical image of each sample: (a) ULM 0.25, (b) ULM 0.50, (c) ULM 0.75, and (d) ULM 1.0.

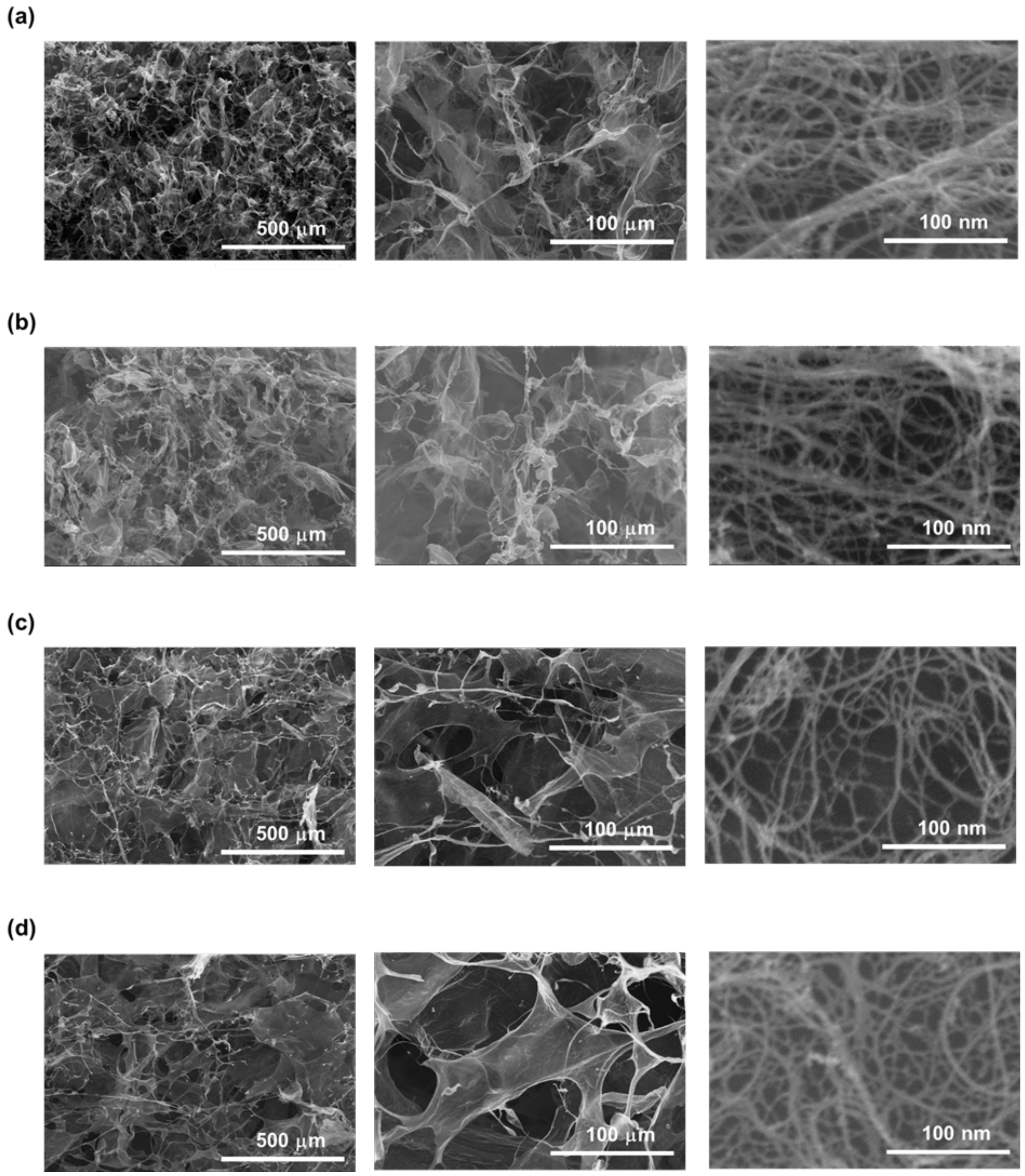

Figure S4. SEM images of the samples: (a) ULM 0.25, (b) ULM 0.50, (c) ULM 0.75, and (d) ULM 1.0.

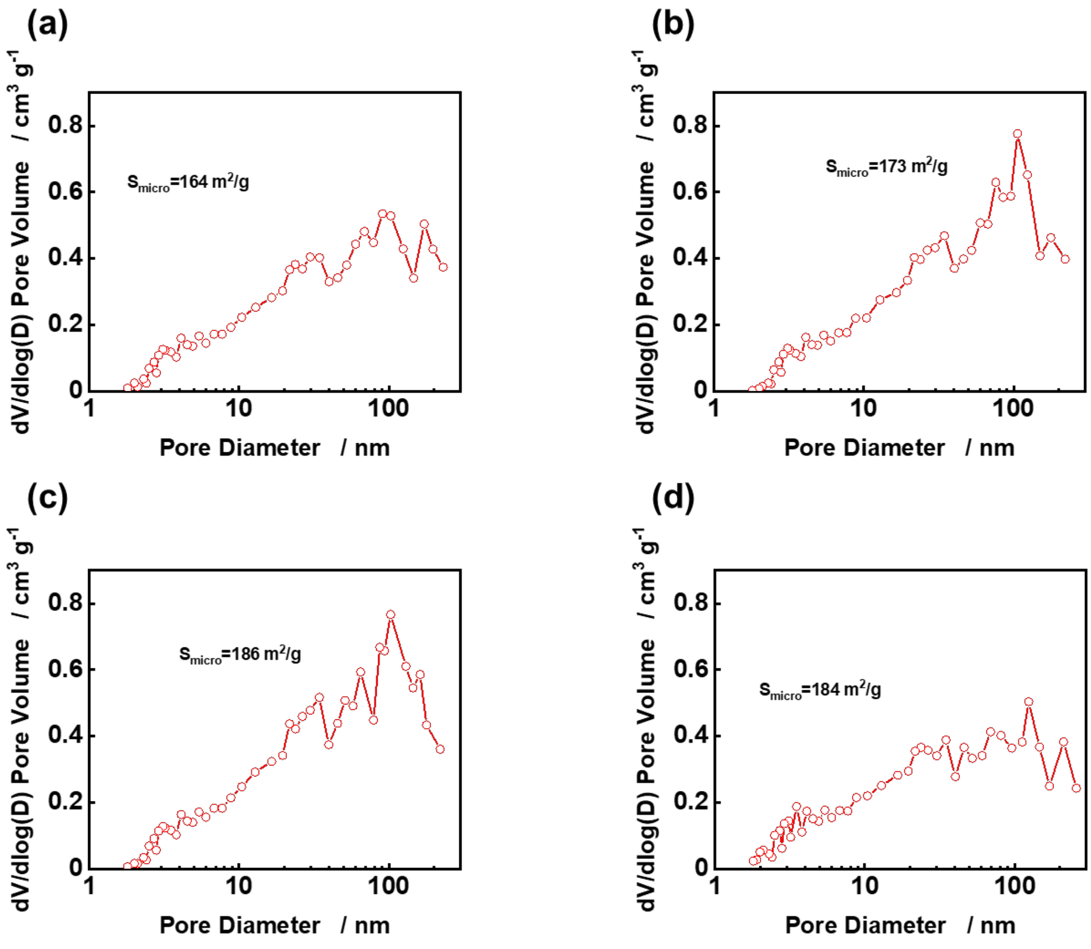

Figure S5. Pore-size distribution and the micropore specific surface area (S_{micro}) of the samples: (a) ULM 0.25, (b) ULM 0.50, (c) ULM 0.75, and (d) ULM 1.0.

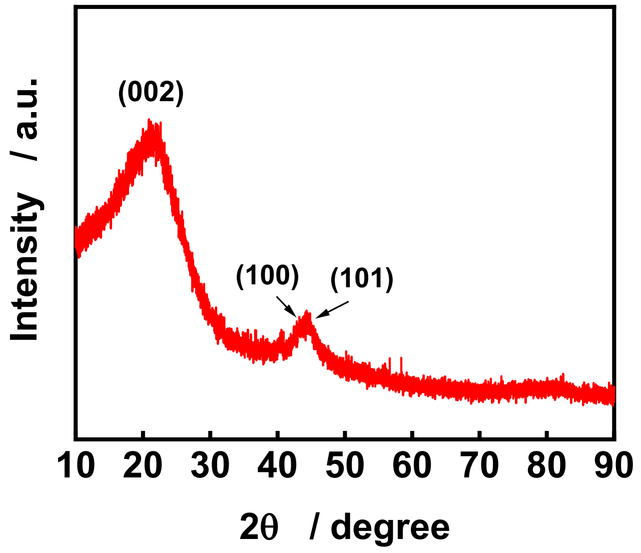

Figure S6. XRD pattern of ULM 1.0.

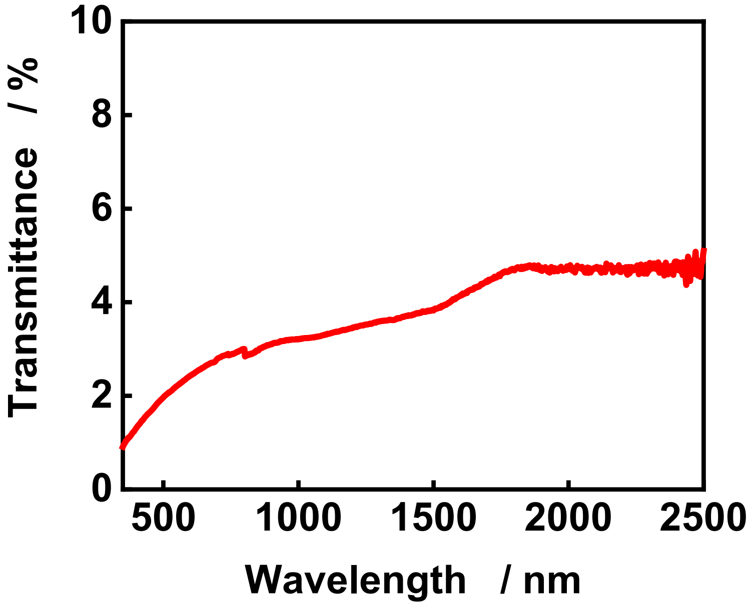

Figure S7. Transmittance of ULM 0.25.

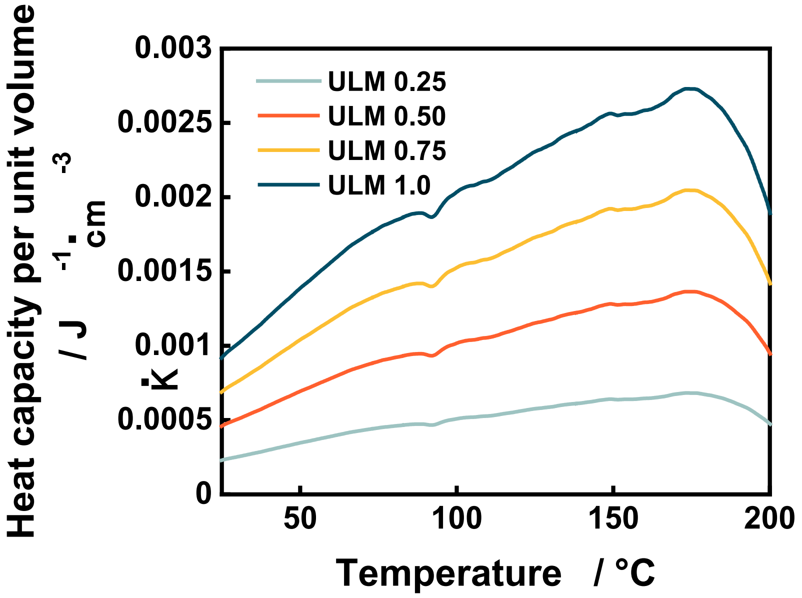

Figure S8. Heat capacity per unit volume of each sample.

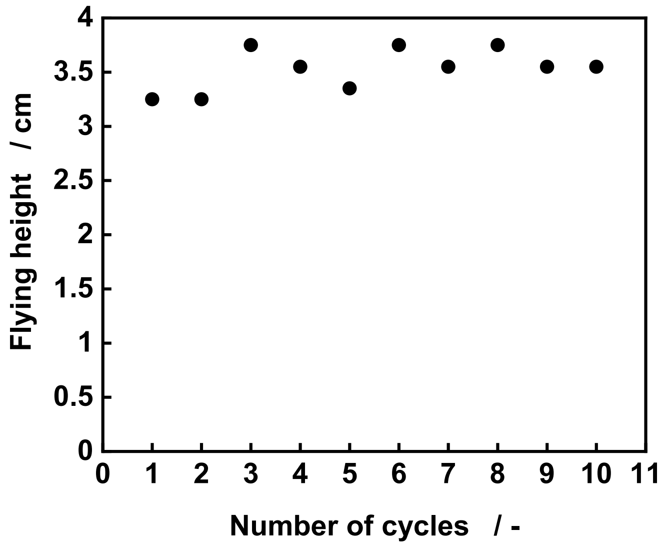

Figure S9. Levitation height of the ultralight aerogel achieved by the ON-OFF cycling of the lamp. The data correspond to ULM 0.75.

Supplementary videos

Videos of the levitation behaviors of the ultralight aerogel were captured in this study. The video speed has not been altered. Videos are of ultralight aerogels with a prepared density of 0.5 mg cm^{-3}.

Video S1. Intermittent levitation of the ultralight aerogel.
Video S2. Touching the levitating aerogel.
Video S3. Response of the ultralight aerogel to the ON-OFF cycling of the lamp.
Video S4. Levitation experiment by sunlight.

Sunlight levitation experiments were conducted by irradiating the aerogel with sunlight focused by a Fresnel lens.

References

1. Sun, W. *et al*. Super black material from low-density carbon aerogels with subwavelength structures. *ACS Nano*. **10**, 9123–9128 (2016).

2. Wang, H. *et al*. artificial trees inspired by monstera for highly efficient solar steam generation in both normal and weak light environments. *Adv. Funct. Mater*. **30**, 2005513 (2020).

3. Fukuda, N., Hatakeyama, M. & Kitaoka, T. Enzymatic preparation and characterization of spherical microparticles composed of artificial lignin and TEMPO-oxidized cellulose nanofiber. *Nanomaterials*. **11**, 917 (2021).

4. DKS Co. Ltd. RHEOCRYSTA. https://www.dks-web.co.jp/product/rheocrysta/index.html (2021).
